# Motor Imagery: A Resource in the Fatigue Rehabilitation for Return-to-Work in Multiple Sclerosis Patients—A Mini Systematic Review

**DOI:** 10.3389/fneur.2021.696276

**Published:** 2021-07-05

**Authors:** Francesco Agostini, Letizia Pezzi, Marco Paoloni, Roberta Insabella, Carmine Attanasi, Andrea Bernetti, Raoul Saggini, Massimiliano Mangone, Teresa Paolucci

**Affiliations:** ^1^Department of Anatomical and Histological Sciences, Legal Medicine and Orthopedics, Sapienza University of Rome, Rome, Italy; ^2^Department of Medical and Oral Sciences and Biotechnologies, University G. d'Annunzio Chieti-Pescara, Chieti, Italy; ^3^Complex Operational Unit (UOC) Physical Medicine and Rehabilitation, Santa Caterina Novella Hospital, Galatina, Italy

**Keywords:** exercise, neurocognitive, cues, rehabilitation, balance

## Abstract

Fatigue is a multidimensional symptom with both physical and cognitive aspects, which can affect the quality of daily and working life activities. Motor Imagery (MI) represents an important resource for use during the rehabilitation processes, useful, among others, for job integration/reintegration, of neurological pathologies, such as Multiple Sclerosis (MS). To define the effective rehabilitation protocols that integrate MI for the reduction of fatigue in patients with MS (PwMS), a literary review was performed through August 2020. Five articles were included in the qualitative synthesis, including two feasibility pilot randomized control trials (RCTs) and 3 RCTs with good quality according to the PEDro score and a low risk of bias according to the Cochrane Collaboration tool. The literature suggested that MI, in association with rhythmic-auditory cues, may be an effective rehabilitation resource for reducing fatigue. Positive effects were observed on perceived cognitive and psychological fatigue. PwMS require greater compensatory strategies than healthy individuals, and the use of rhythmic-auditory cues may be useful for optimizing the cognitive processing of MI, which acts as an internal stimulus that is enhanced and made more vivid by outside cues. These findings provide evidence that MI is a promising rehabilitation tool for reducing fatigue in PwMS and return to work strategies.

## Introduction

Fatigue affects more than 80% of patients with multiple sclerosis (PwMS), among whom 55% report fatigue as being one of the worst symptoms that is experienced, often independently of the level of disability (1). Patients describe fatigue as a feeling of weakness that worsens with exercise or as the day progresses or as an abnormal, constant, and persistent sense of tiredness (2). Fatigue in Multiple Sclerosis (MS) could be a direct effect of the pathological process on the central nervous system (CNS) or secondary to weakness, stiffness, tremor, sleep disturbances, or depression (3, 4). Fatigue management is challenging, and physiotherapy treatment represents a valid resource of fatigue support to complement pharmacological treatment (5, 6). The literature indicates that therapeutic exercise is considered a safe and effective form of rehabilitation for the reduction of fatigue among PwMS and that individualized exercise programs should be designed to address each patient's chief complaint (7). Specifically, endurance and progressive resistance training (PRT) may reduce self-reported fatigue (8, 9). However, in a study by Hameau and colleagues, after a short, intensive, combined rehabilitation program among PwMS, fatigue decreased, but fatigability appeared to increase (10). Fatigue is a multidimensional symptom that involves both physical and cognitive aspects which can affect the quality of daily and working life activities. Often, endurance and aerobic training rehabilitation protocols are not easily applied or well-tolerated among PwMS with medium-to-high levels of disability, such as those patients who require walking or balance aids (11). Some studies focusing on rehabilitation in MS have demonstrated a transitory positive effect on the reduction in fatigue symptoms (7–9, 12); however, other studies that examined the efficacy of various specific rehabilitation programs showed no significant effects on fatigue compared with placebo (13, 14). Novel approaches to physiotherapy in MS include Motor Imagery (MI) and Rhythmic Auditory Stimulation (RAS), which have been shown to improve walking in PwMS, accompanied by reductions in fatigue. Other authors, such as Hanson et al., have suggested that a neurocognitive rehabilitation approach—specifically, the use of MI could represent an important resource for reducing fatigue, because MI involves motor planning and mild exercise execution (15–17). In PwMS, fatigue involves the dysfunction of the circuits connecting the thalamus, basal ganglia, and frontal cortex, which require a specific balance to enable motor and executive motor planning (18–20). MI is the mental rehearsal of movements without actual execution, which involves similar spatial and temporal characteristics, activates the same brain areas that are executed during actual movements (21), and can be performed with or without verbal guidance and additional visual or auditory cues (15, 22). Several studies have investigated the relationship between MS and return-to-work trying to highlight the elements or symptoms that most negatively impact on it, such as fatigue (23). MI represents an important resource for use during the rehabilitation processes, useful, among others, for job integration/reintegration, of neurological pathologies, such as MS (23–25). Several studies have suggested that the connections between rhythmic auditory and motor processing, which reflects sensorimotor synchronization with RAS, may also apply to MI, which involves the mental execution of movements without performing any actual movements (26). The performance of MI has obvious advantages over actual movement practice, including the lack of motor fatigue and reducing the risk of falls, because MI can be realized in a sitting position. In people with other types of neurologic disorders, such as stroke, MI has been shown to improve motor performance (27), with moderate effect sizes (28).

Given the connection between return-to-work and fatigue, and the effects of MI on the latter, the purpose of this mini systematic review was to investigate the effects of rehabilitation protocols that integrate MI to decrease symptoms of fatigue, and therefore, favor the return-to-work, in PwMS.

## Materials and Methods

The Preferred Reporting Items for Systematic Reviews and Meta-Analyses (PRISMA) was used to guide this review (29).

### Data Sources and Search Strategy

The literature research was performed (PubMed, Scopus, PEDro, PsychINFO and Google Scholar) through August 2020 (22), using the following keywords: Job integration/reintegration OR return-to-work AND Multiple sclerosis AND Motor imagery; Multiple sclerosis AND Motor imagery; Multiple sclerosis AND fatigue; Motor imagery AND fatigue; and Multiple sclerosis AND Motor imagery AND fatigue. Two independent reviewers searched each database using the same strategy to ensure proper cross-checking of the results. Table 1 shows the eligibility criteria that were used to determine the inclusion of studies in the review and the algorithm that was developed, based on PICO (patients, intervention, comparison, outcome) (30). The authors evaluated the studies identified by the database searches based on the established inclusion and exclusion criteria (Table 1). The authors independently screened the titles, abstracts, and full texts of all eligible studies. The reference lists of the most relevant studies were scanned for additional citations. Data including the country, author, affiliated institutions, and enrollment periods were extracted and reviewed to identify and exclude duplicate publications using the same cohort. Any disagreements regarding the acceptance of full-text articles were resolved by discussion until a consensus was reached.

**Table 1:** Studies selection criteria and PICO question.

	**Inclusion**	**Exclusion**
Population	PwMS	Other neurological condition
Intervention	Motor Imagery training	Usual treatment
Comparison/control	Usual treatment, PwMS in waiting list, Healthy subjects	
Outcome	Reduction of fatigue / Return-to-work	
Study design	Randomized controlled trial	Other designs, e.g., commentary, opinions, thesis, book chapter, data based on meetings and repositories of dissertations and theses and gray literature.
Other	English language, full text	Other language

*PwMS, people with multiple sclerosis*.

### Quality and Risk of Bias Assessment

The methodological quality of each RCT was assessed using the Physiotherapy Evidence Database (PEDro) scale (31). Two researchers independently applied the scale to each considered study. We considered trials with scores equal to or <9 to be “excellent,” studies, that ranged from 6–8, were considered “good,” trials, that scored 4–5, were deemed to be “fair” quality, and studies, with scores of ≤4, were categorized as “poor” quality (32).

Furthermore, the risk of bias was assessed independently for each study by two authors according to the Cochrane Collaboration's domain-based evaluation framework (33). Main domains were assessed in the following sequence: (1) selection bias (randomized sequence generation and allocation concealment); (2) performance bias (blinding of participants and personnel); (3) detection bias (blinding of outcome assessment); (4) attrition bias (incomplete outcome data, such as that due to dropouts); (5) reporting bias (selective reporting); and (6) other sources of bias. The scores for each bias domain and the final score for the risk of systematic bias were graded as low, high, or unclear risk.

## Results

### Search Results

**Figure 1:**
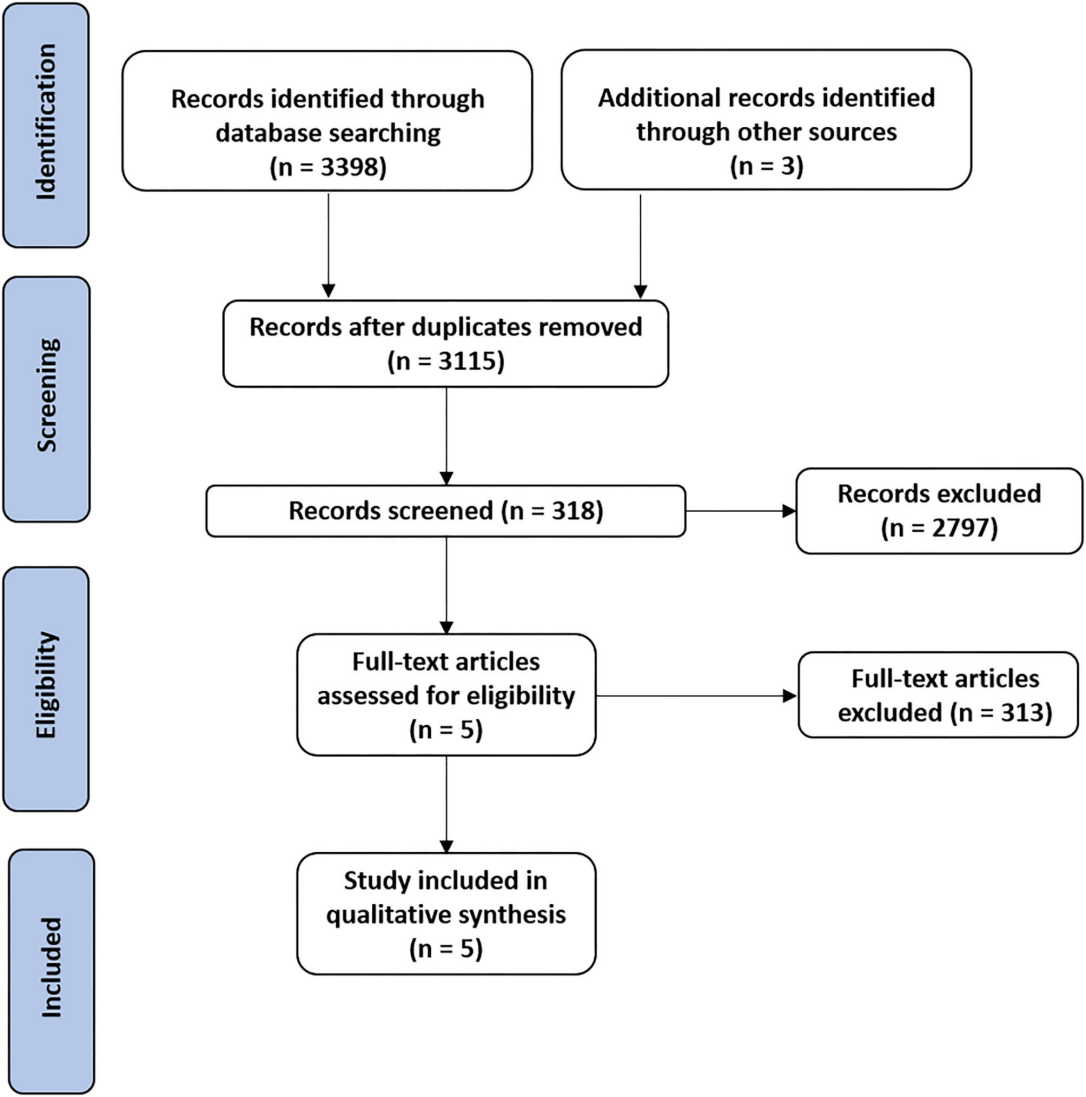
PRISMA flow-diagram showing the selection of the included studies.

The findings are presented in narrative form, including tables and figures, to present the data in a format that is structured around the assessment, sample characteristics, and results. Our initial literature search identified 4,001 records. After removing duplicates, 3,115 records were assessed for eligibility. Following the application of inclusion and exclusion criteria and verifying the full-text articles for eligibility, a total of five articles (34–38) were included in the qualitative synthesis, including two feasibility pilot RCTs and 3 RCTs, as shown in the study flowchart (Figure 1). The mean methodological quality of the five included RCTs, according to the PEDro scale, was 6.8/10 (Table 2), indicating the good overall quality of the included studies. Table 2 also describes the protocols used, the outcomes measured and the times and number of sessions. The risk of bias was considered low for all five studies (Table 3). The most frequent source of potential bias was performance bias, related to the assessments of the blinding of participants and personnel and the blinding of the outcome.

**Table 2:** Summary of the intervention and outcomes (or results) of the included study.

**References**	**Design,** **PEDro** **score**	**Sample** **description,** **age** **(M±SD)**	**EDSS** **(median;** **range)**	**Method**	**Fatigue assessment**	**Outcome measures**	**Timeline/n^**°**^ of session**	**Conclusions**
Seebacher et al. (35)	RCT (Pilot study), 6	TG1 = 10F; 0M (47, 3) TG2 = 7F; 3M (41, 8) CG= 5F; 5M (46.1)	TG1 = 3 (1, 5;4, 5) TG2 =2, 5 (1, 5;4, 5) CG = 2, 5 (1, 5;4, 0)	TG1 = music and verbally cued MI + weekly phone call TG2 = metronome and verbally cued MI+ weekly phone call CG= usual treatment+ weekly phone call	Modified Fatigue Impact Scale	Walking speed and distance (*T25FW; 6-MWT*)	T0 (at baseline) T1 (after 4 week)/n°24	Fatigue reduced in TG1 by median −9.5 (range −31, 5) points, in TG2 by −13 (range −28, 7) points and in CG by −3 (range −17, 4) points.
Seebacher et al. (36)	RCT,7	TG1 = 25F; 9M (43, 8) TG2 = 29F; 5M (45, 4) CG = 31F; 2M (43, 1)	TG1 = 2,0 (1, 5;4, 5) TG2 = 2,0 (1, 5;4, 5) CG = 2,0 (1, 5;4, 5)	TG1 = music and verbally cued MI + weekly phone call TG2 = metronome and verbally cued MI+ weekly phone call CG = usual treatment+ weekly phone call	Modified Fatigue Impact Scale	Walking speed and distance and perception (*T25FW; 6-MWT; MSWS-12*) QoL (*MSIS-29; HRQoL; SF-36; EQ-5D-3L*)	T0 (at baseline) T1 (after 4 week)/n°24	Cognitive and total fatigue reduced significantly in TG1 and TG2. Physical fatigue significantly reduced only in TG1, but psychosocial fatigue did not reduce. There were no clinically meaningful reductions in fatigue.
Seebacher et al. (37)	RCT (Pilot study), 7	TG1 = 4F; 1M (52, 0) TG2 = 5F; 0M (54, 0) TG3 = 4F; 1M 37, 0)	TG1 = 4,5 (2, 0;4, 5) TG2 = 2,5 (2, 5;4, 5) TG3 = 2,5 (1, 5;4, 5)	TG1 = music and verbally cued MI + weekly phone call + usual treatment TG2 = music cued MI + weekly phone call + usual treatment TG3 = non-cued MI + weekly phone call + usual treatment	Modified Fatigue Impact Scale	Walking speed and distance (*T25FW; 6-MWT*) QoL (*MSIS-29)* MI ability (*KVIQ-10; KVIQ-G-10; TDMI*) Sensorimotor sync (gait analysis)	T0 (at baseline) T1 (after 4 week)/n°24	A mild reduction in fatigue was observed in all groups.
Seebacher et al. (38)	RCT, 7	TG1 = 15F; 4M (45, 3) TG2 = 16F; 4M (44, 5) TG3 = 16F; 4M (43, 3)	TG1 = 3,0 (1, 5;4, 5) TG2 = 2,5 (2, 5;4, 5) TG3 = 2, 5 (1, 5;4, 5)	TG1 = music and verbally cued MI + weekly phone call + usual treatment TG2 = music cued MI + weekly phone call + usual treatment TG3 = non-cued MI + weekly phone call + usual treatment	Modified Fatigue Impact Scale	Walking speed and distance (*T25FW; 6-MWT*) QoL (*MSIS-29)* MI ability (*KVIQ-10; KVIQ-G-10; TDMI*) Sensorimotor sync (gait analysis)	T0 (at baseline) T1 (after 4 week)/n°20	Physical and cognitive fatigue and physical QoL significantly reduced only in TG1and TG2 and psychosocial fatigue significantly reduced in all groups (all *p*-values < 0.01).
Kahraman et al. (34)	RCT, 7	TG = 16F; 4M (34, 5) CG = 14F; 1M (36, 0) HCG = 14F; 6M (31, 0)	TG = 1,0 (0;1, 75) CG = 2,0 (0;2, 5) HCG = nv	TG = Telerehabilitation-based MI training CG = waiting list HCG = no treatment	Modified Fatigue Impact Scale Likert scale (0–10)	Gait and balance (*DGI; T25FW; 2-MWT; MSWS-12; TUG; ABC test; posturography*) Cognitive function (*SDMT; SRT; 10/36SRT*) Psychologic and QoL (*HADS; MusiQoL*)	T0 (at baseline) T1 (after 8 week)/n°16	There was a significant reduction from baseline at 8 weeks in the TG, (*p* < 0.05); No significant differences in CG.

*RCT, randomized controlled trial; TG, treatment group; CG, control group; HCG, healthy controls group; T25FW, timed 25-foot walk; 6-MWT, 6-min walking test; MSWS-12, multiple sclerosis walking scale-12; MSIS-29, multiple sclerosis impact scale-29; HRQoL, health- related quality of life; SF-36, short form-36 health survey; EQ-5D-3L, euroquol-5D-3L questionnaire; KVIQ-10, kinaesthetic and visual imagery questionnaire; KVIQ-G-10, kinaesthetic and visual imagery questionnaire – german version; TDMI, time-dependent motor imagery screening test; DGI, dynamic gait index; T25FW, timed 25-foot walk; 2-MWT, 2-min walk test; TUG, timed up and go test; ABC test, activities-specific balance confidence test; SDMT, symbol digit modalities test; SRT, selective reminding test; 10/36SRT, 10/36 spatial recall test; HADS, hospital anxiety and depression scale; MusiQoL, multiple sclerosis international quality of life questionnaire*.

**Table 3:** Risk of bias summary.

Seebacher et al. (36)	Low	+	+	–	–	+	+	?
Seebacher et al. (35)	Low	+	+	–	–	+	+	?
Seebacher et al. (38)	Low	+	+	–	–	+	+	?
Seebacher et al. (37)	Low	+	+	–	–	+	+	?
Kahraman et al. (34)	Low	+	+	–	+	+	+	?
		Random sequence generation	Allocation concealment	Blinding of participants and personnel	Blinding of outcome assessment	Incomplete outcome data	Selective reporting	Other bias

*The ‘+' means low risk of bias; the ‘-' means high risk of bias; the ‘?' means unknown risk of bias. Trials involving three or more high risks of bias were considered as poor methodological quality*.

### Participants

A total of 261 participants were analyzed in the included studies (50 men/211 women) with a median age of 43.55 years. All included studies evaluated a mixed-sex sample, with an Expanded Disability Status Scale (EDSS) score of 2.5, indicating only mild impairments. Fatigue was evaluated using the Modified Fatigue Impact Scale (MFIS) in all included studies (39–41).

### Interventions

Most of the included interventions consisted of home-based, rhythmic, cued MI training (using instrumental music, a metronome, or verbal cueing) (35–38), in which the patients were instructed in the concept of MI and its rehabilitation applications and effects. The patients learned how attention and perception are fundamental components in the planning and controlling of movement before execution. The patients were asked to imagine themselves walking in various manners, accompanied by music and beat, as described by a recent publication (42). Three studies (34, 37, 38) for MI standardization followed the PETTLEP (physical, environmental, task, timing, learning, emotional, and perspective) approach, which may serve as a viable tool to enhance the effectiveness of an intervention. The PETTLEP model is based on neuroscientific findings, developed by Holmes and Collins, and includes a 7-point checklist of guidelines to follow when devising an imagery intervention (43). The durations and intensities of the rehabilitation interventions varied: in 4 studies (35–38), the patients practiced MI for 17 min, 6 times each week for 4 weeks at home. In contrast, Kahraman et al. (34) reported that patients engaged in twice-a-week, 20–30-min sessions for 8 weeks.

### Included Articles

Seebacher et al. (35), with the aim of evaluating changes in fatigue caused by rhythmic motor images, enrolled thirty adults with MS and randomly assigned them into three groups: 17 min of motor imagery, six times a week, for 4 weeks, with music (A) or metronome cues (B) and controls (C). Primary outcomes were recruitment rates, retention, compliance, adverse events, and fatigue (Modified Fatigue Impact Scale). Secondary outcomes were walking speed (25-foot walking time) and distance traveled (6-min walking). The authors concluded that preliminary improvements in walking speed, distance walked, and fatigue of group A need to be confirmed in a larger process.

Seebacher et al. (36), in order to investigate the effect of motor imagery combined with rhythmic cues on walking, fatigue and quality of life in people with MS, enrolled 101 individuals with MS and randomized them into three groups: 17 min of motor imagery, six times a week, for 4 weeks, with musical cues (A) or metronome (B), both with verbal cues, and controls (C). The primary outcomes were walking speed (25-foot timed walk) and distance (6-min walk test). Secondary outcomes were Multiple Sclerosis Walking Scale-12, Modified Fatigue Impact Scale and QoL (Short Form-36 Health Survey, Multiple Sclerosis Impact Scale-29, Euroquol-5D-3L Questionnaire). The authors concluded that rhythm-guided motor images improve walking, fatigue and quality of life in people with MS, while music-guided motor images are more effective.

Seebacher et al. (37), with the aim to obtain preliminary information of changes in walking, fatigue, quality of life (QoL) and MI ability following cued and non-cued MI in pwMS, they enrolled 55 adults with MS and randomized them to three groups: 24 sessions of 17 min of MI with music and verbal cueing (MVMI), with music alone (MMI), or non-cued (MI). Primary outcomes were walking speed (Timed 25-Foot Walk) and walking distance (6-Min Walk Test). Secondary outcomes were recruitment rate, retention, adherence, acceptability, adverse events, MI ability (Kinaesthetic and Visual Imagery Questionnaire, Time-Dependent MI test), fatigue (Modified Fatigue Impact Scale) and quality of life (Multiple Sclerosis Impact Scale-29). The authors concluded that their study suggest that cued and non-cued MI are valuable interventions in patients with MS who were able to imagine movements.

Seebacher et al. (38), with the aim of studying the effects and mechanisms of differently cued and non-cued MI on walking, fatigue and quality of life in patients with MS, enrolled 59 patients with mild to moderate disability and randomized them to music- and verbally cued MI (MVMI), music-cued MI (MMI) or MI. Participants practiced guided or unguided MI of walking for 17 min, six times a week for 4 weeks at home. The primary outcomes were walking speed (timed 25-foot walk) and distance traveled (6-min walk test). The authors concluded that all interventions significantly improved walking. MVMI was superior in improving walking, fatigue and quality of life. The results suggest that MI and sensorimotor synchronization were mechanisms of action.

Kahraman et al. (34), with the aim to investigate the effects of telerehabilitation-based motor imaging training (Tele-MIT) on gait, balance, and cognitive and psychosocial outcomes in people with multiple sclerosis, have created a randomized, controlled pilot trial included people with MS and healthy individuals. People with MS were randomly divided into two groups (intervention and control). The intervention group received Tele-MIT (2/week for 8 weeks). The control group was a wait-list group without any additional specific treatment. Healthy participants served as a baseline comparison. The Dynamic Gait Index, used to assess dynamic balance during walking, was the primary outcome. Secondary outcomes included assessments of walking speed, endurance and perceived ability, balance performance assessed by a computerized posturography device, balance confidence, cognitive functions, fatigue, anxiety, depression, and quality of life. The authors concluded that Tele-MIT is a novel method that proved feasible and effective in improving dynamic balance during walking, walking speed and perceived walking ability, balance confidence, cognitive functions, fatigue, anxiety, depression, and quality of life in people with MS.

## Discussion

The literature reports that MI could represent a rehabilitation resource for relieving symptoms, with the aim of adequate social reintegration and return to work. Evidence suggests that neurocognitive rehabilitation can be used to help patients overcome pain, and MI has been shown to facilitate learning more efficient movement execution strategies to make return to work faster and more manageable by the patients. In PwMS, fatigue represents one of the most disabling symptoms, from a neuromotor point of view, and limiting the execution of activities of daily life and not allowing the patient a complete and timely return to work. This aspect also has consequences from a psychological point of view that led the patient to completely abandon his or her work, no longer feeling able to carry it out. As demonstrated by (44), interventions aimed at reducing fatigue decrease the number of days away from work. The studies that were included in this review showed encouraging results. Catalan et al. have suggested that an MI program could be effective for reducing fatigue in PwMS, with a mean EDSS of 2.5 ± 1.29. The authors observed that patients who were guided by a physiotherapist to correctly perceive kinesthetic information (over a period of 5 weeks of treatment, performed twice a week) learned new motor planning strategies, which might persist up to 6 months after treatment. Seebacher et al. (35–38), in various studies, have reported that MI is an effective rehabilitation resource for decreasing the symptoms of fatigue. The authors used MI in rehabilitation protocols, associated with music and verbal cues, metronomes and verbal cues, or no cues (35–38). Cues are defined as any external stimuli, either temporal or spatial in nature, that are associated with the facilitation of motor activity in PwMS (45). The physical execution of movement and the imagination of movement both involve the activation of similar brain regions (primary motor cortex, supplementary motor area, premotor area, somatosensory area, prefrontal cortex, parietal lobule, cingulate area, basal ganglia, and cerebellum) (46), and various cueing strategies have been associated with improvements in motor performance. The use of cues that are associated with MI can facilitate the process of learning a movement in individuals who present with attention deficits, which is typical of some neurological disorders, including MS (47). The results of (38) are certainly the most interesting as they showed that cued and non-cued MI improved walking speed and walking distance in PwMS, but music- and verbally cued MI were more effective than MI in improve walking, subjective fatigue and QoL (38). In this study, music-cued MI but not MI alone improved fatigue and quality of life while music- and verbally cued MI was more effective, suggesting that these findings are related to the effects of music and verbal cues (38). These results are likely associated with the two important dimensions of fatigue: the perception of fatigue and performance fatigability (48, 49). Differences in these two aspects may explain the discrepancies reported for some rehabilitation approaches to fatigue in MS, in which some authors report increased fatigue after exercise (10, 13, 14), such as the observable decrease in performance during a cognitive or motor task. The subjective perception of fatigue requires a cognitive perspective involving interoception and metacognition (48, 50, 51). The use of music during therapy for neurological diseases may affect cognitive functions, such as increasing verbal memory, in addition to improving motor performance (52–54) and providing benefits for the psycho-emotional sphere (55). The rehabilitative effects of music during therapy for neurological disorders appear to be associated with brain neuroplasticity and neural activation changes; however, the specific mechanisms remain unknown (56). Seebacher et al. (35–38) suggested a 4-week rehabilitation program and identified the specific characteristics of the music cues: the music style and beat were selected based on published summaries of practical guidelines for RAS and other relevant publications (53). The selected music was in 2/4 or 4/4 time, with strong ON and OFF beat patterns, such that every first beat or every first and third beat was stressed. The beat was emphasized by rhythmic verbal cues from the researcher (e.g., rhythmic speech, such as “step-step,” “toe-off”). The music-cued MI synchronizes the motor response, and patients unconsciously adapt their movements to the external rhythm (56), which has been shown to be well-suited for improving gait during rehabilitative protocols, as reported by Seebacher. The patients enrolled in these studies reported the perception that the treatment was safe and convenient, and even those enrolled in non-cued-MI arms reported satisfaction with the intervention, especially in terms of the focus on body awareness, without distraction (37). Generally, the studies by Seebacher and colleagues on the use of MI combined with rhythmic-auditory cues have suggested that this approach resulted in positive effects on perceived cognitive fatigue and various aspects of walking among PwMS. The synchronization between external rhythmic signals and movement showed positive effects compared with the isolated use of MI during rehabilitation (35–38). The study by Hereman et al. (57) showed that visual stimuli improved the spatial accuracy of movements during MI, whereas auditory stimuli improved temporal precision, both of which had positive effects on the vividness of the images. This finding suggested that cues related to movement may facilitate the generation of MI, and the use of external stimuli to provide the spatial and temporal components of the movement appeared to improve the efficacy of MI. PwMS require compensatory strategies to overcome their movement dysfunction, and the use of cues has been shown to be useful for optimizing the cognitive processing required for MI (58), which acts as an internal stimulus that is enhanced and made more vivid by outside cues. Moumdjian et al. (59, 60) compared the abilities of PwMS with those of healthy controls (HC) for sustaining synchronization of a 12-min period of walking accompanied by music and a metronome. They analyzed physical and cognitive fatigue, motivation, and gait compared with walking in silence. PwMS could walk for 12 min of uninterrupted walking under all tested conditions; however, improved synchronization, reduced perception of cognitive fatigue, and high motivation were observed when external cues were used. Listening to music instead of a metronome might be more pleasurable and may increase adherence to the MI rehabilitation process, which is important for home-based interventions. Moreover, music may be an interesting form of diversifying the training (61) and could have positive effects on fatigue during therapeutic treatment with MI. The study by (34) described training in tele-motor imagery (MIT), conducted by an expert physiotherapist. At the beginning of the session, the authors proposed relaxation exercises, including 5 min of free breathing, followed by deep breathing and awareness exercises. To evoke MI, the physiotherapist used auditory, visual, tactile, and olfactory cues that were easily available within the patient's home context. Authors used multimodal cues for enhancing the motor imagery vividness. In contrast to the studies from Seebacher et al. (35–38), these cues were not real but imagined. Patients in the MIT-treated group reported functional improvements in fatigue. Telerehabilitation was reported to be effective for the treatment of various neurological conditions, including MS. Telerehabilitation reflects a new approach to facilitate the delivery of rehabilitation programs in the patient's home, using new technologies (62). However, a Cochrane review highlighted the limitations and the paucity of high-quality studies conducted in PwMS to date. MS is a complex and challenging condition requiring individualized and integrated multidisciplinary care, and telerehabilitation interventions are difficult to standardize (63, 64). Several studies have demonstrated that mental practice through MI can result in motor improvements, indicating that MI represents a potential tool for motor learning, relearning, and rehabilitation, especially among people with physical disabilities (63). Mental practice with MI offers the opportunity to improve motor skills through safe and self-paced training among people with severe disabilities, such as PwMS, and the association of MI with auditory cues appears to improve outcomes. The evidence currently present in the literature on the use of MI of PwMS to reduce fatigue, although not numerous, suggests how this method can be effective not only for an improvement in the quality of life and autonomy in the activities of daily life, but also in conclusion, for a better return to work, not only by imagining work tasks (as a kind of imaginary occupational therapy), but also because patients can do it at home even after working.

### Strengths and Limitations

According to our knowledge, this is the first review on the use of MI, for the reduction of fatigue in PwMS, aimed at return to work. This certainly represents a current and extremely important issue today. Our work is not free from limitations such as certainly the low number of works included which is secondary to the lack of study and scientific evidence present in scientific literature today.

## Conclusion

Fatigue in PwMS is a complex clinical problem, with a lack of currently effective treatments and it represents one of the most severe restrictions on return to work in PwMS. Therefore, when establishing a rehabilitation plan, particular attention should be paid to the most convenient techniques, aimed at a better and faster *restitutio ad integrum* of the patient and a more effective return to work. MI could be a promising rehabilitation tool, which has been shown to be effective for decreasing the symptoms of fatigue and improving motivation. These findings provide evidence that MI is a promising rehabilitation tool for reducing fatigue in PwMS and return to work strategies. Given the potential benefits of MI for neurological rehabilitation, we recommend future studies to explore the motor representations in PwMS to improve the provision of effective and tailored rehabilitative treatments.

## Author Contributions

FA, LP, and TP: conceptualization. MP and RS: methodology. MM: software, data curation, and project administration. MP and AB: validation. CA and TP: formal analysis. FA: investigation. TP and AB: resources. FA, LP, and MP: writing—original draft preparation. AB: writing—review and editing. FA: visualization. RS and TP: supervision. All authors contributed to the article and approved the submitted version.

## Conflict of Interest

The authors declare that the research was conducted in the absence of any commercial or financial relationships that could be construed as a potential conflict of interest. The reviewer GC declared a shared affiliation, with no collaboration, with the authors FA, MP, RI, AB, and MM at the time of the review.
